# Supplementary material for: UV decontamination of personal protective equipment with idle laboratory biosafety cabinets during the COVID-19 pandemic
Source: PLoS One. 2021 Jul 26;16(7):e0241734. doi: 10.1371/journal.pone.0241734 (PMC8312969; doi:10.1371/journal.pone.0241734)
Supplement: S1 File — (PDF) [file pone.0241734.s001.pdf]

## S1 File

### Supplemental Methods

#### *Photodiode measurements*

Photodiodes were operated in zero-bias photovoltaic mode. While the photodiodes had a wide UV spectral range, we did not utilize filters since the diodes were only used to measure relative irradiance and we expected UVC output to be a stable fraction of total UV output from the bulbs.

Since voltage measured (less than 1V) was substantially less than the saturating voltage of approximately 4.8V, we expect to be operating within the linear dynamic range of the photodiodes. The photodiodes used in measurements were of the same model number and from the same lot and were therefore expected to have the same operating characteristics. Measurements of light intensity from the photodiodes were recorded by a Raspberry Pi at 40ms intervals for a total period of 4 seconds. A circuit board with an LM324N operational amplifier (for signal amplification) and an ADS1015 analog-to-digital converter were used to interface the photodiodes and the Pi (**Figure S1**).

We affixed three photodiodes (MTPD4400D-1.4) to a standard N95 respirator (3M) and measured UV fluence from nine positions (across a 3x3 grid) equally spaced on the counter of each BSC (**Figure S2**).

Resulting data were used to generate heatmaps of the values from all three photodiodes and the UV meter at each position of the 3x3 grid at the base of the BSCs. Analysis was performed in the R programming language [1] using the ggplot2 [2] and dplyr [3] packages (all code and data may be viewed in the github repository [4]).

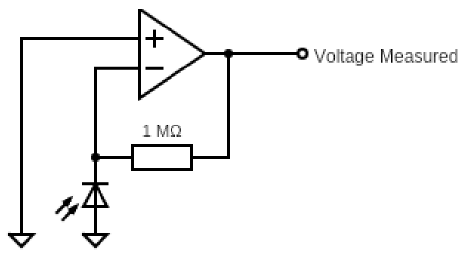


**Supplemental Figure S1. Schematic of part of the circuit containing the photodiode.** A 1 megaohm resistor was chosen to sufficiently amplify the signal from the photodiode. The resulting voltage (less than 1V) was substantially less than the saturating voltage of approximately 4.8V. Voltage measurements were made with an analog-to-digital converter connected to a Raspberry Pi but could also be read through an oscilloscope.

#### *Fit and filtration testing*

The N95 respirators were cut into 70 mm $\times$ 70 mm pieces and tested in a circular acrylic air duct with an inner diameter of 50 mm. Ambient aerosols were loaded as the pollutant source. The number concentrations of 0.3 - 1 $\mu$m particles were measured by an optical particle counter (Aerotrak 9306, TSI Inc., USA). The concentrations were record every 1 min for 2 times upstream the respirator filter and then 2 times downstream. The single-pass filtration efficiency $\eta$, which is a function of particle size diameter, $d_p$, was calculated

by:

$$\eta(d_p) = \left(1 - \frac{C_{down}(d_p)}{C_{up}(d_p)}\right) \times 100\% \tag{4}$$

where $C_{up}$ and $C_{down}$ are the particle number concentrations (pcs/L) at upstream and downstream of the respirator filter, respectively, and each a function of ($d_p$). The pressure drop across the respirator filter was measured by a differential gauge. The air temperature, relative humidity, and filtration velocity were measured by an airflow/temperature meter at the air duct exhaust.

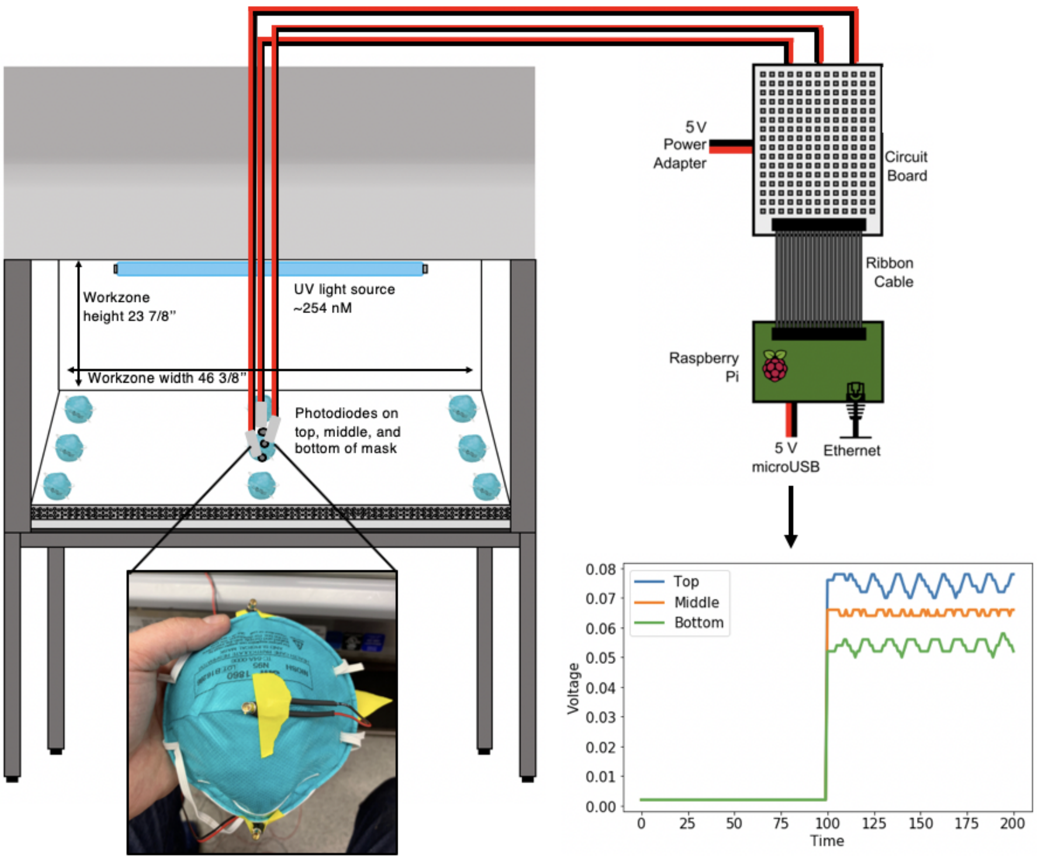


**Supplemental Figure S2. Schematic of our process for measuring light intensity across the base of a BSC with photodiodes.** A photodiode was attached to the top (north), middle, and bottom (south) of an N95 mask, and the voltage of light that reached diodes was measured both with the UV light turned off and then on. This measurement was performed within each sector of a 3x3 grid at the base of the BSC workzone as illustrated.

## Supplemental Physical Results

Using an array of photodiodes attached to a standard N95 mask (see **Supplemental Figure S2**), we assayed the heterogeneity due to mask geometry at different positions along the bottom of the cabinet (**Supplemental Figure S3**). The median proportional variance across each mask was 1.42 between the highest and lowest intensities. If we limit our consideration to the front corners of the array (the areas that receive the lowest irradiance), the median proportional variance across each mask was 2.17 (indicating a higher variance due to mask geometry in these areas).

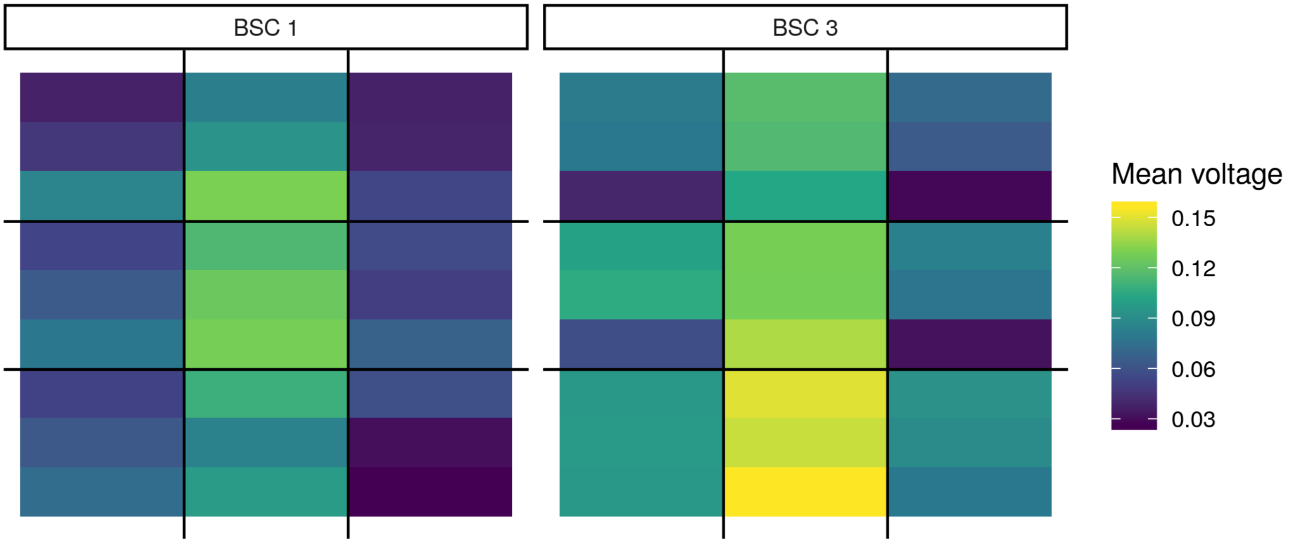


**Supplemental Figure S3. Relative UV intensity as a function of position across the base of two BSCs.** Each of the nine sections per BSC shows the voltage from three photodiodes attached to the surface of an N95 mask (top, middle, and bottom). The three photodiodes were attached to different positions on the mask (**Supplemental Figure S2**) to demonstrate UV differences across mask surface. To account for ambient light, voltages recorded with the UV lights off were subtracted from the voltages recorded with the UV lights on.

## Supplemental Results: Filtration testing

| No | Mask | Effi-0.3μm | Effi-0.5μm | Effi-1μm | ΔP (pa) | $V_{air}$ (m/s) | T (°C) | RH (%) |
|---|---|---|---|---|---|---|---|---|
| #1 | 3M1860S-0 | 90.2% | 90.0% | 95.6% | 185.8 | < 0.1 | 23.7 | 21.5 |
| #2 | 3M1860S-15min | 92.1% | 92.9% | 95.6% | 186.1 | < 0.1 | 23.6 | 22.1 |
| #3 | 3M1860S-30min | 93.2% | 93.7% | 94.6% | 186.3 | < 0.1 | 23.6 | 22.7 |
| #4 | 3M1860S-1h | 92.9% | 92.7% | 94.8% | 184.8 | < 0.1 | 23.6 | 22.5 |
| #5 | 3M1860S-2h | 93.0% | 93.5% | 95.6% | 187.2 | < 0.1 | 23.7 | 22.0 |
| #6 | 3M1860S-6h | 89.6% | 90.5% | 92.9% | 186.6 | < 0.1 | 23.7 | 21.7 |
| #7 | 3M1860S-days on/off | 93.1% | 93.9% | 94.3% | 186.3 | < 0.1 | 23.6 | 22.1 |

**Supplemental Figure S4. UV irradiation at the doses discussed does not adversely affect mask filtration efficiency for particles of size 0.3, 0.5, or 1 micron.**

## Supplemental Results: Additional Virologic Experiments

In the intervening months in which the COVID pandemic has somewhat subsided in the US, and laboratories have reopened, we endeavored to reproduce these results in a secondary laboratory environment. To this end, we performed normalizing UV measurements in the biosafety cabinet of a neighboring infection control laboratory (Donskey lab, Cleveland Louis Stokes VA). These measurements can be seen in **Supplemental Figure S5**.

| 57 cm from bulb | | |
|---|---|---|
| Back | | |
| 35 uW/cm$^2$ | 44 uW/cm$^2$ | 38 uW/cm$^2$ |
| 91 uW/cm$^2$ | 117 uW/cm$^2$ | 94 uW/cm$^2$ |
| 102 uW/cm$^2$ | 150 uW/cm$^2$ | 130 uW/cm$^2$ |
| Bulb | | |
| Front | | |

| 34.3 cm from bulb | | |
|---|---|---|
| Back | | |
| 25 uW/cm$^2$ | 36 uW/cm$^2$ | 26 uW/cm$^2$ |
| 104 uW/cm$^2$ | 157 uW/cm$^2$ | 108 uW/cm$^2$ |
| 247 uW/cm$^2$ | 427 uW/cm$^2$ | 308 uW/cm$^2$ |
| Bulb | | |
| Front | | |

| 19 cm from bulb | | |
|---|---|---|
| Back | | |
| 19 uW/cm$^2$ | 26 uW/cm$^2$ | 22 uW/cm$^2$ |
| 164 uW/cm$^2$ | 218 uW/cm$^2$ | 174 uW/cm$^2$ |
| 394 uW/cm$^2$ | 590 uW/cm$^2$ | 420 uW/cm$^2$ |
| Bulb | | |
| Front | | |

**Supplemental Figure S5. UV irradiation as measured in the Donskey lab biosafety cabinet.**

Using the methods described herein, we calculated the required time and position within these secondary cabinets and performed decontamination experiments using a protocol adapted from ASTM standard E3179-18 "Determining Antimicrobial Efficacy of Ultraviolet Germicidal Irradiation against Influenza Virus on Fabric Carriers with Simulated Soil." Whole 3M 8210 N95 respirators inoculated in triplicate with PHI6 from stock with no additional organic load added. Ten 1ul drops inoculated on the interior surface, strap, and exterior surface, and the nose clip then allowed to dry 1 hour. Mask treated with UV-C in hood with mask placed 19 cm from the UV-C bulb in the center of the hood for 76 minutes on each side of the mask. Mask pieces were then punched out and placed in 15 mL flat bottom tubes. 1 mL of PBS added, samples vortexed on high for 1 minute, then serial and pour plated using a double agar overlay method. Treated test samples compared against untreated controls. In these experiments we saw 2-4 log reductions on all parts of the mask tested (range 2.23 to 3.96 log reduction), see **Supplemental Figure S6**.

| Controls | Recovered ($Log_{10}$ PFU) | Average | Tests | Recovered ($Log_{10}$ PFU) | Average | Reduction ($Log_{10}$ PFU) |
|---|---|---|---|---|---|---|
| Control Interior | 3.00 | 3.43 | Interior | 2.00 | 0.67 | 2.76 |
| | 3.30 | | | 0.00 | | |
| | 4.00 | | | 0.00 | | |
| Control Exterior | 3.08 | 3.46 | Exterior | 0.00 | 0.00 | 3.46 |
| | 3.26 | | | 0.00 | | |
| | 4.04 | | | 0.00 | | |
| Control Strap | 4.60 | 4.56 | Strap | 3.30 | 2.33 | 2.23 |
| | 4.60 | | | 2.00 | | |
| | 4.48 | | | 1.70 | | |
| Control Nose Clip | 5.30 | 4.67 | Nose clip | 0.30 | 0.71 | 3.96 |
| | 4.70 | | | 1.83 | | |
| | 4.00 | | | 0.00 | | |

**Supplemental Figure S6. Effective decontamination was observed on multiple aspects of the masks tested using proposed protocol.**

This was subsequently repeated with non-porous face shields, with the same protocol, except using 30 seconds of exposure. This was completed side by side with 7% ethanol wipe down. There was no virus recoverable after the UV decontamination or ethanol cleaning, see **Supplemental Figure S7**.

| Controls | Recovered ($Log_{10}$ PFU) | Average | Reduction ($Log_{10}$ PFU) |
|---|---|---|---|
| Control Interior | 5.30 | 5.46 | N/A |
| | 5.48 | | |
| | 5.60 | | |
| UV-C Treatment | 0.00 | 0.00 | 5.46 |
| | 0.00 | | |
| | 0.00 | | |
| 7-% Ethanol Wipe | 0.00 | 0.00 | 5.46 |
| | 0.00 | | |
| | 0.00 | | |

**Supplemental Figure S7. No virus was recovered from a non-porous face shield after either 30 second UV decontamination or 7% ethanol wash.**

This was subsequently repeated in MS2 phage with only single log reduction. This is not unexpected given the known reduction is UVGI efficacy in non-enveloped viruses. [5, 6] These data are available in the github repository.

## References


1. Team, R. C. *R: A Language and Environment for Statistical Computing*. R Foundation for Statistical Computing, Vienna, Austria (2019).
2. Wickham, H. *ggplot2: Elegant Graphics for Data Analysis* (Springer-Verlag New York, 2016).
3. Wickham, H., François, R., Henry, L. & Müller, K. *dplyr: A Grammar of Data Manipulation* (2020). R package version 0.8.4.
4. Dolson, E. & Krishnan, N. Data and analysis for use of uv-c radiation in idle biosafety cabinets to sterilize personal protective equipment, DOI: 10.5281/zenodo.3724005 (2020). URL: https://doi.org/10.5281/zenodo.3724005.
5. Cadnum, J. L. *et al.* Effectiveness of ultraviolet-c light and a high-level disinfection cabinet for decontamination of n95 respirators. *Pathogens and Immunity* **5**, 52 (2020).
6. Kayani, B. J. *et al.* Uv-c tower for point-of-care decontamination of filtering facepiece respirators. *American journal of infection control* **49**, 424–429 (2021).
