# Supplementary material for: UV decontamination of personal protective equipment with idle laboratory biosafety cabinets during the COVID-19 pandemic
Source: PLoS One. 2021 Jul 26;16(7):e0241734. doi: 10.1371/journal.pone.0241734 (PMC8312969; doi:10.1371/journal.pone.0241734)
Supplement: Attachment — Submitted filename: Response to Reviewers - PONE-D-20-33184R1.pdf [file pone.0241734.s002.pdf]

Cleveland Clinic
Department of Translational Hematology and Oncology Research
9500 Euclid Avenue, Cleveland Ohio, 44195, US
May 23, 2021
e-mail: scottj10@ccf.org

Dear PLoS ONE Editorial Team,

We thank you and Drs. Ylitalo and Fornace for the generally positive review of our work, and for the opportunity and recommendations to improve it through revisions. To this end, we would like to resubmit a revision of our manuscript (PONE-D-20-33184) with a slightly changed title: "UV Sterilization of Personal Protective Equipment with Idle Laboratory Biosafety Cabinets During the COVID-19 Pandemic." Please find appended a point by point response to the thoughtful and constructive comments from the reviewers. Please note that all changes will appear in blue text in the revised document. As we work in LaTeX, these are not tracked, *per se*, but can be changed back and forth from "blue" (changed) to "black" (clean), by altering line 99 in the code. We have submitted pdf version of both clean and changed.

We apologize for the delay in returning this manuscript, doing the experiments requested by the reviewers took more time than anticipated due to laboratory closures during the pandemic. This further necessitated adding two extra authors, Dr. Curtis Donskey, an infection control and UV decontamination expert, and a member of his laboratory, Miss Jennifer Cadnum. While we were unable to provide perfect validation based on our constraints, we believe that the extra data provided strengthen our result, and answer the requests of the reviewers.

The final point we would like to raise is that we felt somewhat torn in how to approach revisions: this paper was undertaken during a period of historic stress on the scientific community. What we presented is the effort of multiple labs, in multiple states, doing what they could together to answer a pressing question. If this study were to be done again, from scratch, in 'normal situations', it would have been done differently. But it wasn't. It was done at the peak of the COVID pandemic in the US, and we have chosen to maintain as much of the original narrative as possible.

Many thanks again for your time and efforts,

Jacob Scott, on behalf of all coauthors

Review response

Here, we will respond to each reviewer in turn. Comments from reviewers will be in *italics*, and the subsequent comments in **bolded plain text**. Where reviewers make un-numbered comments, we will respond in paragraph form, and where they are numbered we will respond in an enumerated fashion.

Reviewer #1:

This study validates the use of the UVC lamp that is standard in the back of biosafety cabinets for filtering facepiece respirator (FFR also referred to as N95 respirator) decontamination. The authors did a nice job quantifying the radiation dose within the space of the cabinet. They also provided guidance to the required exposure time to achieve acceptable decontamination. In addition, the authors validated UVGI as method to decontaminate face shields. The equations to calculate minimum exposure time were provided as well. The authors conclude that the biosafety cabinets that are common in microbiology labs are an effective method to decontaminate respirators. Overall, the study was well organized and followed the scientific method. The method and results were clearly described. However, additional work is needed in order to provide value to the reader. I will address the limitations of this study next.

Many thanks for these positive comments.

1. *Novelty: using biosafety cabinet UV light to decontaminate FFR was established by NIOSH/NPPTL lab (Viscousi et al., 2007 and 2009) in multiple publications, see for example reference 5 of this paper. This study provides more details on the method already established by Viscousi et al..*

   We recognize that the use of UVGI to decontaminate masks is not entirely novel, but are glad that the reviewer sees the novelty of our contribution.

2. *Method: cutouts of N95 respirators are not a good representation of a respirator. Decontamination for the purpose of reuse should be validated for the entire respirator which is three dimensional and has additional parts such as headbands and nose clip. Shadowing effect from headbands and respirator curvature were not studied. Headband placement during decontamination is key to ensure headbands are not covering parts of the respirator, thereby, preventing decontamination of those parts. The authors need to address headband placement during the process. One type of respirator was evaluated: 3M 1860 cup, this is one of several different geometries that include pleated and folded respirators. How does the shape of those models affect UVGI performance as disinfection method? I realize shortages of N95 respirators is a constraint, so the authors may not have access to other respirator geometries.*

   Thank you for these comments. The reviewer is 100% correct in their points, and we have added a short section in the discussion to bring up these limitations and made certain to address headband placement in the protocol. The reviewer is correct that we did not/do not have access to other respirator geometries, however, in a separate publication we have looked at others using UVGI (not biosafety cabinets). This publication is now referenced. As to the cutouts not being representative of N95 masks, we do agree, however the protocol used was a standard one in the infection control literature, and so for scientific rigor, we keep this method.

3. *Typically, most of filtered pathogens (viruses and bacteria) are deposited in the filter layer of a respirator, not on the coverweb (top surface). N95 respirators have electrically charged filter that captures pathogens, while the coverweb does not have that property, therefore, most pathogens (and particles) end up in the inner filter layers of a respirator. The authors of this study deposited droplets of virus inoculum on the top surface of the respirator cutout. This method does not simulate real use conditions, and validating virus deactivation on the surface layer does not correlate with full respirator decontamination. The authors indicate in the limitations section of the paper that virology results for the inner layers of the respirator are pending, I recommend waiting to include those results before publication as virus viability on the inner layers is significant since most filtered virus will be deposited in those inner layers.*

   We agree with the reviewer in many ways, but to maintain standard and rigor, we follow the standard protocols as laid down in ASTM standard E3179-18 "Determining Antimicrobial Efficacy of Ultraviolet Germicidal Irradiation against Influenza Virus on Fabric Carriers with Simulated Soil."

4. *Decontamination of face shield: it is not clear to me that UVGI is appropriate or practical to decontaminate face shields. I suggest the authors compare the efficacy of UVGI with soap and water rinse and disinfectant wipes. The key property that should be maintained after decontamination is the antifog performance of the face shield. Does UVGI maintain the antifog performance? How does it compare to washing with soap and water or using disinfectant wipes? Do those alternate methods adversely affect antifog performance of face shields?*

   Thank you for this comment. In lieu of further testing, we have now added this to the discussion, and further discussed work from the Armani lab, in which face masks were the sole focus.

5. *Statistical analysis: at least three replicates per experiment should be conducted. It was not clear if replicates were done and how many, no error bars are shown on the graphs.*

   This is a good spot. At the time of first submission, all laboratories except a very few were closed in the US. Therefore, the experimental results we showed were all we could manage at the time (not triplicate). Subsequently, as labs have reopened, we sought to generalize our results. Now, in the supplements, we present a number of well conducted experiments supporting our findings. We were torn, editorially, as to how to proceed – and we ended by keeping the original narrative of the paper as a 'moment in time', and added the new experiments to the supplements.

6. *Need clarification: the literature recommendation of 1 J/cm2 exposure was cited. However, the authors did not clarify weather this is the total exposure (both sides of the respirator) or required exposure dose per side. Since a respirator is a three dimensional opaque device, to achieve effective disinfection it is necessary to expose outer and inner surfaces to the UV radiation.*

   Both sides require exposure, we have made sure to highlight this in the manuscript throughout.

Reviewer #2:

This reviewer (also scientific editor for this manuscript) was favorable to publication of this report in PloSONE. Shortage of PPE's during the covid pandemic is a serious problem and the authors have

shown that standard UV bulbs, which are used for sterilizing laminar flow hoods, are effective in inactivating the virus. Such equipment is commonly available. Single strand viruses are particularly sensitive to UV radiation but this needed to be confirmed in COVID-19 virus which the authors have accomplished. The authors have done a reasonable job on dosimetry and results are convincing. Potential problems with this approach are addressed such as aging of typical 254 nm UV bulbs. While they have discussed the challenge of the 3 dimensional nature of some PPE equipment and the issue of UV radiation penetration into filter material, these issues need to be elaborated on further.

Many thanks for aknowledging the utility of this simple method, and our best efforts at dosimetry and discussion of the limitations. We have elaborated in the discussion on the points requested by this reviewer.
